# CNN-BiLSTM and DC-IGN fusion model and piecewise exponential attenuation optimization: an innovative approach to improve EEG emotion recognition performance

**DOI:** 10.3389/fncom.2025.1589247

**Published:** 2025-06-16

**Authors:** Shaohua Zhang, Yan Feng, Ruzhen Chen, Song Huang, Qianchu Wang

**Affiliations:** College of Information Science and Technology, Xizang University, Lhasa, China

**Keywords:** EEG emotion recognition, DC-IGN, piecewise exponential decay strategy, CNN, BiLSTM

## Abstract

EEG emotion recognition has important applications in human-computer interaction and mental health assessment, but existing models have limitations in capturing the complex spatial and temporal features of EEG signals. To overcome this problem, we propose an innovative model that combines CNN-BiLSTM and DC-IGN and fused both outputs for sentiment classification via a fully connected layer. In addition, we use a piecewise exponential decay strategy to optimize the training process. We conducted a comprehensive comparative experiment on the SEED and DEAP datasets, it includes traditional models, existing advanced models, and different combination models (such as CNN + LSTM, CNN + LSTM+DC-IGN). The results show that our model achieves 94.35% accuracy on SEED dataset, 89.84% on DEAP-valence, 90.31% on DEAP-arousal, which is significantly better than other models. In addition, we further verified the superiority of the model through subject independent experiment and learning rate scheduling strategy comparison experiment. These results not only improve the performance of EEG emotion recognition, but also provide new ideas and methods for research in related fields, and prove the significant advantages of our model in capturing complex features and improving classification accuracy.

## 1 Introduction

In recent years, affective computing, as an important research direction in the field of artificial intelligence, has been widely concerned (Li et al., 2023). Emotion are an important part of human cognition and interaction, playing a key role in decision making, perception, and interpersonal interaction (Alarcao et al., 2019). However, in traditional computer interaction, it is often difficult for machines to understand human emotional states, resulting in a lack of human interaction experience. To remedy this shortcoming, affective computing has emerged, aiming to identify a user’s emotional state by measuring their physiological signals, thus enabling more natural and intelligent human-computer interaction. Traditional psychophysiological signals, such as voice (Khalil et al., 2019), facial expression (Li and Deng, 2022) and text (Alswaidan et al., 2020), can provide emotional information, but these signals are easily interfered by external factors, such as voice changes, facial camouflage, cultural differences, etc., resulting in low accuracy and reliability of emotion recognition. In contrast, electroencephalography (EEG), as a non-invasive, portable and easy-to-use physiological signal acquisition technology, is able to directly reflect the neural activity of the cerebral cortex, which is closely involved in the production and regulation of emotions. Therefore, by analyzing EEG signals, the intrinsic changes of human emotions can be captured more accurately, with higher recognition accuracy and reliability. EEG emotion recognition technology has shown wide application prospect and important research significance in many fields. First, in terms of emotional health care, the technology can not only help diagnose and treat emotional disorders such as depression and anxiety by identifying emotional states, but also assist in monitoring and evaluating treatment effects and guiding personalized treatment programs. Second, in the field of personalized human-computer interaction, EEG emotion recognition makes intelligent assistants, games, and virtual reality experiences more human, capable of providing customized services and interactive experiences based on the user’s emotional state. In addition, in multimedia content recommendation, the technology can recommend content that is more in line with the needs of users according to their emotional states, and improve the user experience. In neuroscience research, EEG emotion recognition provides a new perspective to explore the cognitive mechanism of human emotion. Finally, in other fields such as education and transportation, EEG emotion recognition can also help optimize teaching strategies and prevent traffic accidents, bringing more convenience and well-being to human life. To sum up, EEG emotion health care and personalized human-computer interaction, but also has extensive application potential in multimedia content recommendation, neuroscience research and many other fields.

Traditional EEG emotion recognition methods mainly include time domain analysis, frequency domain analysis (such as power spectral density analysis and frequency band energy calculation), time frequency analysis (such as short-time Fourier transform and wavelet transform), and machine learning algorithms (such as support vector machines, artificial neural networks, etc.). These methods identify emotional states by extracting specific features of EEG signals, such as amplitude, frequency, and time-related distributions of brain waves. However, the limitations of these methods are that they tend to rely on specific frequency bands and brain regions, are difficult to capture complex emotional changes, and are susceptible to noise interference, resulting in poor recognition accuracy. In addition, these methods usually require a large amount of training data and computational resources, and lack adaptability to individual differences, limiting their widespread popularization in practical applications.

With the development of deep learning techniques, researchers began to explore the use of deep neural networks to improve the accuracy and efficiency of EEG emotion recognition. Zhong et al. (2020) proposed a regularized graph neural network (RGNN) model to address three challenges in EEG emotion recognition tasks: underutilization of EEG signal topology, cross-subject EEG variation, and label noise. The model used a biologically-supported adjacency matrix to capture the channel relationships in EEG signals, and improved the robustness of the model through two regularization methods, NodeDAT and EmotionDL. A large number of experiments on SEED and SEED-IV datasets showed that RGNN model outperformed the existing baseline model in both agent-related and agent-free classification setting, and revealed the relationships between key brain regions and channels related to emotion recognition through neuronal activity analysis. Tao et al. (2023) studied how to use EEG signals for emotion recognition. In order to solve the problem that traditional methods needed to manually design features and it was difficult to extract more discriminative features, this paper proposed an ACRNN model, which integrated channel attention and extended self-attention mechanism, and could effectively extract spatial and temporal features from original EEG signals, and achieved better recognition accuracy than other methods. Cheng et al. (2020) studied how to use multi-channel electroencephalogram (EEG) data for emotion recognition. Traditional sentiment recognition methods required manual feature extraction, while deep neural networks, although better, required a large amount of training data and complex hyperparameter settings. In order to overcome these shortcomings, this paper proposed a deep forest (gcForest) based emotion recognition method. In this method, the original EEG signals were first preprocessed by baseline removal, then the data was mapped to a two-dimensional frame sequence, and the spatial and temporal information was extracted by the scanning module of gcForest and the cascade forest module, respectively, and finally the emotion classification was performed. The experimental results showed that the proposed method was more accurate than the existing method on two open databases DEAP and DREAMER, and it was not sensitive to parameter setting, and could also achieve good performance on small-scale training data. Yin et al. (2020) studied how to use EEG signals for emotion recognition. This paper proposed a deep learning-based emotion recognition method, which combined graph convolutional neural network (GCNN) and long short-term memory network (LSTM). GCNN was used to extract graph domain features from EEG signals, and LSTM was used to extract temporal features. Experimental results showed that the proposed method achieved high emotion recognition accuracy on DEAP dataset, which was superior to other traditional machine learning models and deep learning models. Li et al. (2021) proposed the BiDANN model, which combined left and right hemisphere information and domain adversarial learning techniques to improve the accuracy of EEG emotion recognition. In addition, in order to solve the problem of emotion recognition across EEG of subjects, a BiDANN-S model was proposed to reduce the influence of individual differences on recognition results by introducing a discriminator. Experimental results on SEED database showed that BiDANN and BiDANN-S model achieved better accuracy than existing methods in EEG emotion recognition tasks.

Although various models for EEG emotion recognition have been proposed in the literature, they have some limitations. For example, the RGNN model in Zhong et al. (2020), while taking into account the topology and cross-subject variation of EEG signals, could be computationally expensive when dealing with large-scale data. The ACRNN model in Tao et al. (2023) improved feature extraction by introducing attention mechanisms, but might be sensitive to specific EEG bands, thus limiting its ability to generalize in different emotional states. Although the gcForest method in Cheng et al. (2020) did not require large amounts of training data and complex hyperparameter setting, it might be less efficient in processing high-dimensional EEG data. The GCNN-LSTM model in Yin et al. (2020) combines spatial and temporal features, but might not be efficient enough at fusing information from different modes. The BiDANN model in Li et al. (2021), although considering left and right hemisphere information and domain adversarial learning, might have some limitations in processing EEG sequences with multiple time steps. In contrast, out model is able to process EEG data from six time steps simultaneously, which helps to capture the dynamic process of emotional change, thus improving the accuracy of classification. With the CNN-BiLSTM structure, we can effectively extract the spatial and temporal features of EEG signals, while DC-IGN further enhances the ability to learn the underlying emotional features. The full connection layer fuses the output of CNN-BiLSTM and DC-IGN. This integrated learning method can synthesize the advantages of the two models and improve the overall classification performance. The piecewise exponential decline helps balance the convergence rate and model stability during training, ensuring that the model can still be optimized in the later stages of training.

Our main contributions are as follows:

1. In data processing, the model uses six input tensors of shape (8, 9, 4) to represent EEG data at different time steps, replacing the traditional single-time-step processing. After feature extraction by CNN-BiLSTM and DC-IGN respectively, the outputs of these two features are fused by a fully connected layer for emotion classification. This approach can better capture the dynamic process of emotional changes, combine the spatio-temporal feature extraction capabilities of CNN-BiLSTM and the probabilistic modeling capabilities of DC-IGN, and enhance the diversity of features and the accuracy of classification.
2. The model introduces a Deep Convolutional Inverse Graph Network (DC-IGN) based on the Variational Autoencoder (VAE). During the encoding process, it performs probabilistic modeling on features, using CNN and DNN for encoding and decoding, respectively. This generates more robust feature representations, enhances the model’s ability to handle complex EEG signals, improves the classification accuracy and generalization ability, and breaks through the limitations of traditional single-feature extraction methods.
3. When training the model, a piecewise exponential decay strategy is used to adjust the learning rate. Different from the traditional fixed learning rate or single-decay strategies, this strategy dynamically adjusts the learning rate according to the training process. It ensures that the model converges quickly in the early training stage and maintains stable and accurate optimization in the later stage, avoiding premature convergence and overfitting, and effectively improving the training efficiency and the final classification performance.
4. Multiple sets of experiments were conducted on the SEED and DEAP datasets, including model comparisons, learning rate strategy comparisons, and subject-independent experiments. These experiments fully demonstrate the superiority of our model and provide valuable references for future research on emotion classification.

The chapters are distributed as follows. Chapter 1 gives a brief background and related introduction. Chapter 2 introduces feature extraction. Chapter 3 introduces the model structure. Chapter 4 makes experimental analysis. Chapter 5 provides a brief summary.

## 2 Feature extraction

**Figure 1:**
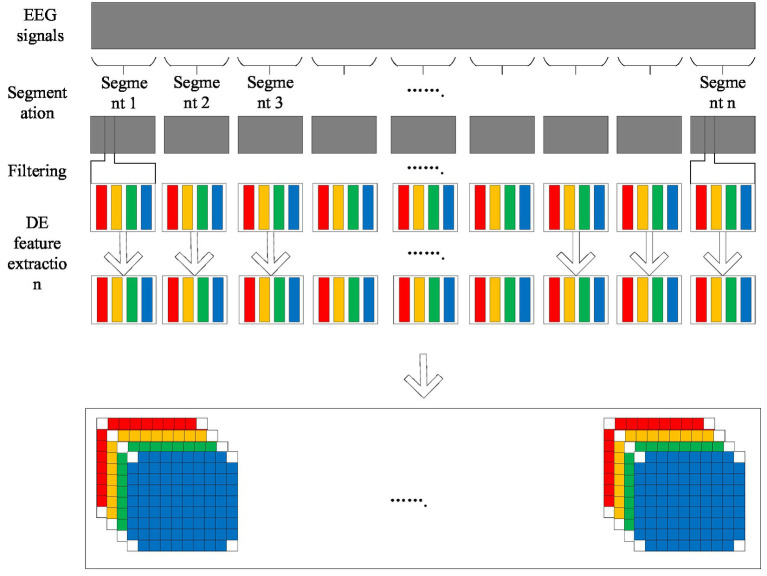
4D feature organization.

Electroencephalogram (EEG) signals contain a wealth of information, which can reflect the activity pattern of the brain in different states. Emotion is a complex brain state, and its characteristics are also reflected in EEG signals. Traditional emotion recognition methods tend to focus on the characteristics of EEG signals in time domain or frequency domain, ignoring the information in space and time dimension. In order to capture emotion features more comprehensively, this paper adopts an EEG emotion recognition method based on 4D feature organization (Figure 1) (Fangyao et al., 2020).

The 4D feature organization method extracts the frequency, spatial and temporal information of EEG signals through the following steps:

Segmentation: The original EEG signal is divided into multiple segments of fixed length, each segment corresponds to an emotion label.

Filtering: Bandpass filtering is performed on each segment to extract information in specific frequency bands, such as the Theta, Alpha, Beta and Gamma bands.

The differential entropy (DE) feature extraction for each band of EEG signals can quantify the complexity and randomness of EEG signals, and its calculation formula is (Equation 1)


(1) DE=−∑p(x)∗log(p(x))

Spatial representation: The DE feature vector of each frequency band is converted into a two-dimensional distribution map, where each pixel corresponds to the DE feature value of one electrode.

The two dimensional maps of different frequency bands are stacked together to form a three dimensional feature map, which is expanded in the time dimension to form a four dimensional feature structure. The structure contains four dimensions, namely frequency, space, time and fragment, which can describe emotional characteristics more fully.

4D feature structure can effectively integrate the frequency, space and time information of EEG signals, and provide richer feature representation for emotion recognition tasks, thus improving the accuracy of emotion recognition. In addition, the 4D feature structure is also well interpretable and can help researchers better understand the neural mechanisms of emotions.

## 3 Model structure

The model accepts six input tensors, each representing one time step of EEG data in the shape of (8,9,4).

In the context of electroencephalogram (EEG) data analysis, the use of six input tensors can be theoretically grounded in the understanding of the temporal and spatial characteristics of EEG signals. EEG signals are dynamic and reflect the electrical activity of the brain over time. The brain’s neural activity changes continuously, and different time steps capture different states of this activity. By using six input tensors, each corresponding to a distinct time step, the model can analyze the evolution of the EEG signal across multiple moments.

Temporally, the six-time-step approach allows the model to account for short-term and medium-term changes in the brain’s electrical patterns. For example, certain cognitive processes or emotional states may manifest as transient changes in the EEG signal that occur over a few milliseconds to seconds. Each time step captures a snapshot of these changes, and by considering multiple time steps together, the model can better understand the temporal dynamics underlying the EEG data. Spatially, the shape of each tensor (8,9,4) implies that the EEG data is being represented in a multi-dimensional space. The three dimensions can represent different aspects of the EEG recording, such as different electrode locations on the scalp, frequency bands of the EEG signal, and other physiological or mathematical features. The combination of multiple time steps with this spatial representation enables the model to analyze how the spatial patterns of EEG activity change over time.

During pre-processing to mitigate inter-subject variability in signal amplitude and baseline noise. Each subject’s EEG data is normalized using their own mean and standard deviation, reducing bias introduced by individual physiological differences (e.g., skull thickness or electrode impedance). Second, we integrate adaptive feature recalibration within the CNN-BiLSTM framework. This involves dynamically adjusting feature importance weights based on subject-derived metrics (e.g., spectral power distribution or connectivity patterns) during training. The DC-IGN module further augments this by modeling subject-invariant latent representations through probabilistic disentanglement, separating shared emotional features from idiosyncratic noise. Additionally, we evaluate the model on a stratified subject-independent split, ensuring that each fold in cross-validation contains mutually exclusive subjects. This rigorously tests the model’s ability to generalize to unseen individuals. To quantify individual variability, we analyze per-subject performance metrics.

The process of deriving these six input tensors from EEG data typically involves several pre-processing steps:

EEG data is collected using an EEG recording device, which consists of multiple electrodes placed on the scalp. These electrodes detect the electrical activity of the brain and convert it into voltage signals. The signals are then amplified and digitized at a certain sampling rate (e.g., 256 Hz or 512 Hz), resulting in a time-series of voltage values for each electrode.

The continuous EEG time-series data is segmented into non-overlapping or overlapping time windows. Each time window corresponds to a single time step. For example, if the sampling rate is 256 Hz and we want to have time steps of 1 s, each time step will contain 256 data points for each electrode. In the case of six input tensors, six consecutive time windows are selected from the segmented data.

For each time step, the data is further processed to extract relevant features. This may involve operations such as filtering to remove noise and artifacts, and transforming the data into different frequency domains (e.g., using the Fast Fourier Transform). The shape (8,9,4) of the tensor may be obtained by grouping electrodes into subsets, calculating different frequency - band features, and applying other feature-engineering techniques. For example, the 8 might represent different groups of electrodes, the 9 could correspond to different frequency bands, and the 4 could represent other statistical or physiological features calculated from the EEG data within each electrode - frequency - band combination.

After feature extraction, the data for each time step is organized into a tensor of shape (8,9,4). Each element in the tensor represents a specific feature value corresponding to a particular spatial and frequency combination within that time step. Finally, the six tensors corresponding to six consecutive time steps are fed into the model as input.

**Figure 2:**
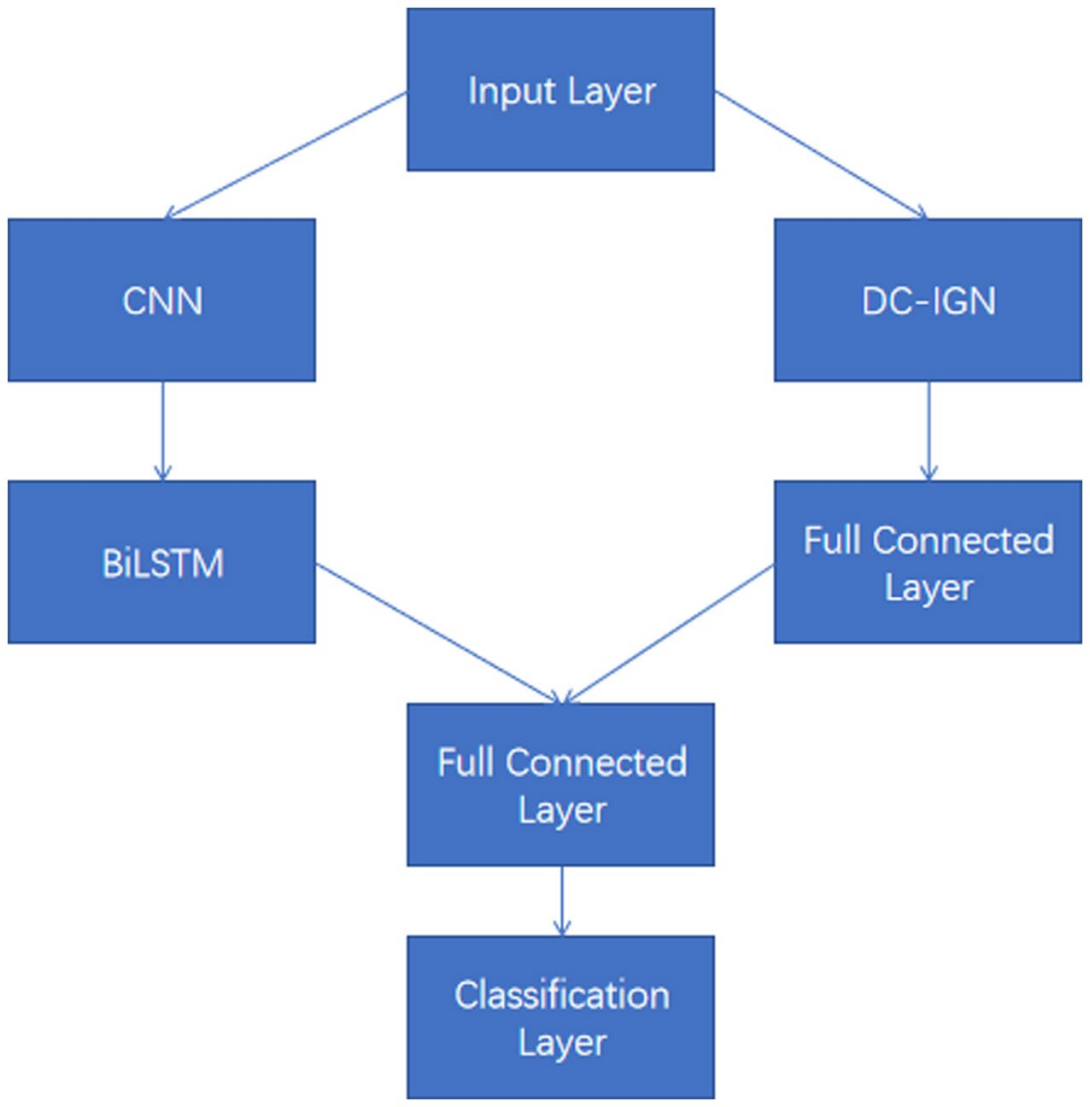
Model structure diagram.

**Figure 3:**
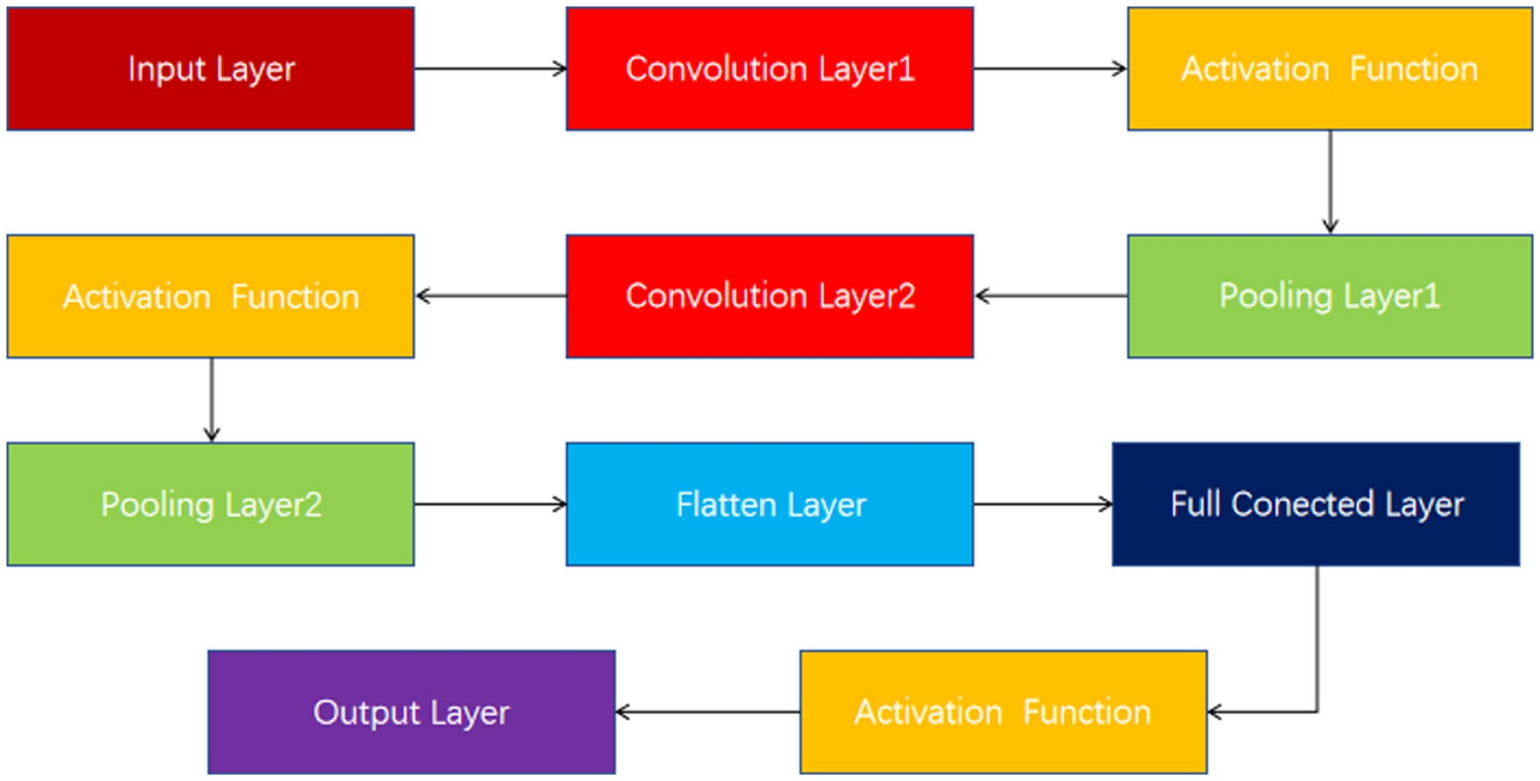
CNN structure.

These input tensors capture changes in the EEG signal over different time and spatial dimensions. The model structure diagram is shown in Figure 2. For each input tensor, spatial features are first extracted by convolutional neural network, and then temporal dynamic features are captured by bidirectional long short-term memory network. CNN is able to extract local spatial features efficiently, while BiLSTM is able to capture temporal dependencies in the sequence. DC-IGN is used for probabilistic modeling of features so that complex transformations and generation of data can be learned. After the CNN-BiLSTM and DC-IGN modules, the respective output results are fed into the full connection layer for further feature fusion and processing. These fully connected layers are responsible for integrating features from different modules to generate a final representation for sentiment classification. The fused features are fed into a fully connected layer, and the results of emotion classification are output through a softmax layer. This part is responsible for predicting the corresponding emotional categories based on the extracted and fused features. In the training process, the learning rate strategy of piecewise exponential decay is adopted to help the model adjust the learning rate in different periods of training, so as to better converge and optimize. CNNs (Lecun et al., 1998), or Convolutional Neural networks, are a popular deep learning model that is particularly good at processing data with grid-like structures, such as images and videos. By learning the spatial hierarchical representation of the input data, CNN can automatically extract the features in the data, thus achieving remarkable success in image recognition, target detection, speech recognition and other fields. The core idea of CNN is to automatically extract the features of the input data through the convolutional layer, then reduce the dimension of the features through the pooling layer, and finally through the full connection layer for classification or other tasks. The main components of CNN include convolution layer, activation function, pooling layer and fully connected layer (Figure 3).

The convolutional layer is the basis of the CNN, which simulates the sensitivity of neurons to specific regions in biological visual systems. The convolution operation computes the features of a local region by sliding a small learnable filter (also known as a kernel or convolution kernel) over the input data.

Assuming that the input is a two-dimensional matrix X and the filter is a small two-dimensional matrix W, the convolution operation can be expressed as (Equation 2):


(2) $$\;(X*W)(i,j)=\sum_{m=0}^{k-1}\sum_{n=0}^{k-1}X(i+m,j+n)W(m,n)\;$$

Where, k is the size of the filter, usually 3 × 3 or 5 × 5.

For color images, the input is usually three channels (RGB), so the filter also needs to have the corresponding three channels. The convolution operation becomes (Equation 3):


(3) $$\left(X*W)(i,j)=\sum_{c=0}^{2}\sum_{m=0}^{k-1}\sum_{n=0}^{k-1}X_{c}(i+m,j+n)W_{c}(m,n\right)$$

Where, c indicates the channel index.

The result of a convolution operation is usually Rectified Linear Unit (ReLU) to increase the nonlinear expressibility of the model (Equation 4).


(4) ReLU(x)=max(0,x)

The pooling layer is used to reduce the size of the feature map and reduce the amount of computation while preserving the main features. The most common is MaxPooling, which slides a window on the feature map and takes the maximum value in the window as output (Equation 5).


(5) $$\text{MaxPooling}(X)(i,j)=\max_{m=0}^{p-1}\max_{n=0}^{p-1}X(i\times p+m,j\times p+n)$$

Where *p* is the size of the pooled window, usually 2 × 2.

The fully connected layer flattens the previously extracted features, connects them to one or more fully connected neural network layers, and finally classifies them through the softmax layer.

A typical CNN consists of multiple convolution layers, activation functions, pooling layers, and fully connected layers. The output of each layer acts as the input to the next layer, progressively extracting more abstract features.

An important feature of CNN is weight sharing. In the convolution layer, the same filter is applied to the entire input feature map, which means that the parameters of the filter are shared at different locations. This greatly reduces the number of parameters in the model and reduces the risk of overfitting.

Bidirectional Long Short Term Memory (BiLSTM) (Zhou et al., 2016) is a neural network model widely used in sequential data processing. It inherits the advantages of LSTM and improves the understanding of sequence data by considering both forward and reverse information of sequence.

The LSTM is the basis for BiLSTM, a special type of recurrent neural network (RNN) designed to solve the problem of disappearing gradients or exploding gradients that traditional RNNs encounter when dealing with long sequences. LSTM controls the flow of information by introducing a memory unit (cell state) and three gating structures (forget gate, input gate, output gate).

Forget gate: determines what information is discarded from the cell state (Equation 6).


(6) $$f_{t}=\sigma (W_{f}[h_{t-1},x_{t}]+b_{f})$$

Input gate: determines what new information is stored in the cell state (Equations 7–9).


(7) $$i_{t}=\sigma (W_{i}[h_{t-1},x_{t}]+b_{i})$$

(8) $$\tilde{C_{t}}=\tanh(W_{C}[h_{t-1},x_{t}]+b_{C})$$

(9) $$C_{t}=f_{t}\times C_{t-1}+i_{t}\times \tilde{C_{t}}$$

Output gate: determines which information in the cell state is output as a hidden state (Equations 10, 11).


(10) $$o_{t}=\sigma (W_{o}[h_{t-1},x_{t}]+b_{o})$$

(11) $$\;h_{t}=o_{t}\times \tanh(C_{t})$$

Where σ represents the sigmoid function, tanh is the hyperbolic tangent function, and W and b are the weight matrix and the bias term, respectively.

**Figure 4:**
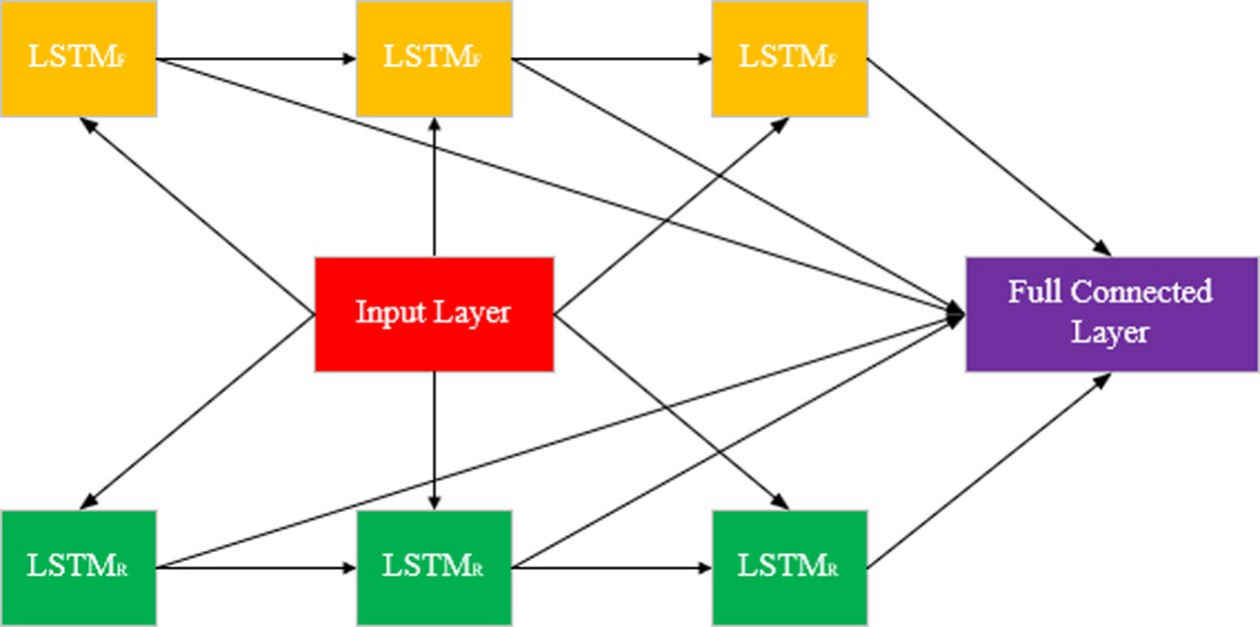
BiLSTM structure.

BiLSTM obtains more comprehensive sequence information by feeding the sequences forward and backward into two separate LSTM networks, and then combining the outputs of the two (Figure 4). In BiLSTM, the input sequence is fed into both forward LSTM and reverse LSTM. Forward LSTM: The data is processed from the beginning to the end of the sequence. Reverse LSTM: Processing data from the end of the sequence to the beginning. The output of BiLSTM can be combined with the output of forward and reverse LSTM in a number of ways.

Concatenation: The stitching together of the hidden states of the forward and reverse (Equation 12).


(12) $$h_{t}=\text{concat}(\vec{h_{t}},\overset{\leftarrow }{h_{t}})$$

Summation: The sum of the forward and reverse hidden states (Equation 13).


(13) $$h_{t}=\vec{h_{t}}+\overset{\leftarrow }{h_{t}}$$

Averaging: Averaging forward and backward hidden states (Equation 14).


(14) $$h_{t}=\frac{\vec{h_{t}}+\overset{\leftarrow }{h_{t}}}{2}$$

$\vec{h_{t}}$ and $\overset{\leftarrow }{h_{t}}$ indicate the hidden state of forward and reverse LSTM in time step t, respectively.

VAE is a powerful generative model that generates new data samples by learning the latent representations of data distribution (Doersch, 2016). It consists of two parts. The encoder and the decoder. The encoder maps the input data to the latent variable space and learns the distribution of the latent variables. The decoder maps the latent variables back to the data space and learns to generate the data distribution.

The goal of VAE is to maximize the objective function, which consists of two parts: reconstruction loss and KL divergence, respectively measuring the quality of the input data reconstructed by the decoder and the difference between the latent variable distribution and the prior distribution learned by the encoder. The formula for maximizing the objective function of VAE is as follows (Equation 15):


(15) $$L=E_{z}\sim Q[\text{logP}(X|z)]-D[Q(z|X)\|P(z)]$$

The first term $E_{z}\sim Q[\text{logP}(X|z)]$ is the reconstruction loss. It measures the quality of the input data X reconstructed by the decoder. Ideally, the decoder should be able to accurately reconstruct the input data, minimizing the reconstruction loss as much as possible. The second term, D[Q(z∣X)‖P(z)] , is the KL divergence. It measures the difference between the latent variable distribution Q(z|X) learned by the encoder and the prior distribution P(z). Ideally, the encoder should learn the latent variable distribution as similar as possible to the prior distribution, making the KL divergence as small as possible.

**Figure 5:**
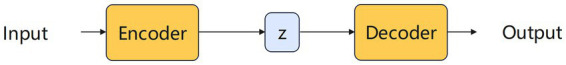
VAE model diagram.

The structure diagram of the VAE model is shown in the Figure 5. The working principle of VAE is to encode the input data into the latent variable space, and then sample and decode from the latent variable space to generate new data samples. By minimizing the objective function, VAE learns a generative model that is capable of generating new data samples similar to the training data. VAE has extensive applications in fields such as image generation, text generation, audio generation, data dimension reduction and anomaly detection.

A deep convolutional inverse graph network (DC-IGN) (Kulkarni et al., 2015) is a deep learning model designed to learn interpretable and decoupled representations from images. It uses a deep convolutional and deconvolution architecture, combined with a variational autoencoder (VAE) framework, to break down an image into multiple independent potential variables, such as pose, illumination, and shape, allowing for flexible control of image generation.

Multi-layer convolution layer and pooling layer are used to extract image features and map them to potential variable space. The latent variable space contains multiple independent latent variables, each representing a specific aspect of the image, such as pose, lighting, or shape. The output of the encoder is used to parameterize the posterior distribution q(z|x) of the latent variable, where q is selected as the multivariate normal distribution, expressed as (Equation 16):


(16) $$q(z|x)=N(y_{e};\mu_{z},\sum_{z})$$

Here, y_e_ is output of the encoder, μ_z_ and Σ_z_ are the mean and variance of the underlying variable z, respectively.

The underlying variables are reconstructed into images using multiple deconvolution layers and upper sampling layers. The decoder can produce an image similar to the original image and is able to flexibly edit the image according to changes in the underlying variables. The decoder converts the latent variable z to the image x, which is (Equation 17):


(17) x′=decoder(z)

DC-IGN’s underlying variables represent decoupage features of the image, such as pose, lighting, and shape. Each latent variable is only sensitive to a specific aspect of the image, such as the pose latent variable is only sensitive to the pose of the image, and the illumination latent variable is only sensitive to the illumination of the image. The latent variable space contains multiple independent latent variables, each of which represents a specific aspect of the image. The decoupling between the underlying variables allows the model to flexibly control the generation of the image, for example by changing the pose, lighting, or shape.

The DC-IGN model in this paper adopts a symmetrical five-layer encoder-decoder architecture. The encoder consists of 5 layers of convolutional networks (each layer uses 5 × 5 convolutional kernels, with a step size of 2, and the number of channels is 64–128–256-512-1024 in sequence). After compressing the input image into a feature map of 7 × 7 × 1,024, the mean *μ* and variance *σ* of the 256-dimensional latent variables are output through the fully connected layers. The decoder corresponds to a 5-layer deconvolution network (with the number of channels being 1,024–512–256-128-64), and uses convolution kernels of the same size to gradually upsample and reconstruct the image.

## 4 Experimental analysis

### 4.1 Dataset

The SEED dataset (Zheng and Lu, 2015) is an EEG emotion recognition dataset collected and published by the BCMI Laboratory of Shanghai Jiao Tong University. The dataset contained 15 movie clips covering positive, negative and neutral emotions, each about 4 min long. The dataset recorded EEG signals from 15 subjects, each of whom performed two experiments, for a total of 30 experiments. The data set is preprocessed, including downsampling, filtering and segmentation, and provides a variety of feature extraction methods, such as differential entropy, power spectral density, etc. The dataset can be used to study EEG based emotion recognition algorithms and models, and promote the development of the field of emotion computing.

DEAP (Dataset for Emotion Analysis using Physiological Signals) (Koelstra et al., 2011) is a widely used dataset for studying the relationship between physiological signals and human emotions. This dataset contains a variety of physiological signals recorded from 32 subjects while watching video clips, including electroencephalography (EEG), electrical skin activity (EDA), heart Rate (HR), and Respiration rate (Respiration Rate). They also collected the subjects’ subjective emotional ratings of each video, such as valence and arousal. DEAP data sets have important application value in the fields of affective computing, human-computer interaction and mental health, providing researchers with rich multi-modal physiological data and corresponding affective labeling, so as to explore and develop more accurate emotion recognition algorithms and systems.

### 4.2 Analysis of experimental results

In this paper, the performance evaluation of EEG emotion recognition is usually quantified by classification accuracy (Acc) (Equation 18) and its standard deviation (Std) (Equation 19), and the calculation formula is:


(18) $$\mathrm{Acc}=\frac{\mathrm{TP}+\mathrm{TN}}{\mathrm{TP}+\mathrm{TN}+\mathrm{FP}+\mathrm{FN}}$$

(19) $$\mathrm{Std}=\sqrt{\frac{1}{N-1}\sum_{i=1}^{N}(x_{i}-\bar{x}}{)}^{2}$$

Where TP, TN, FP, FN represent true positives, true negatives, false positives and false negatives respectively, and $\bar{x}$ denotes the mean accuracy across *N* = 5 trials (5-fold cross-validation), and $x_{i}$ is the accuracy of the i−th fold. For deeper model interpretation, we employed t-SNE dimensionality reduction to visualize feature distributions and analyzed class-specific performance through confusion matrices.

We design a series of experiments to verify the effects of different model structures on the emotion classification task. CNN is used to extract local features from EEG data, LSTM, BiLSTM, and BiGRU are used to capture different time dependencies of time series data, respectively, while DC-IGN, as part of variational autoencoders (VAE), enhances feature representation through probabilistic modeling.

**Figure 6:**
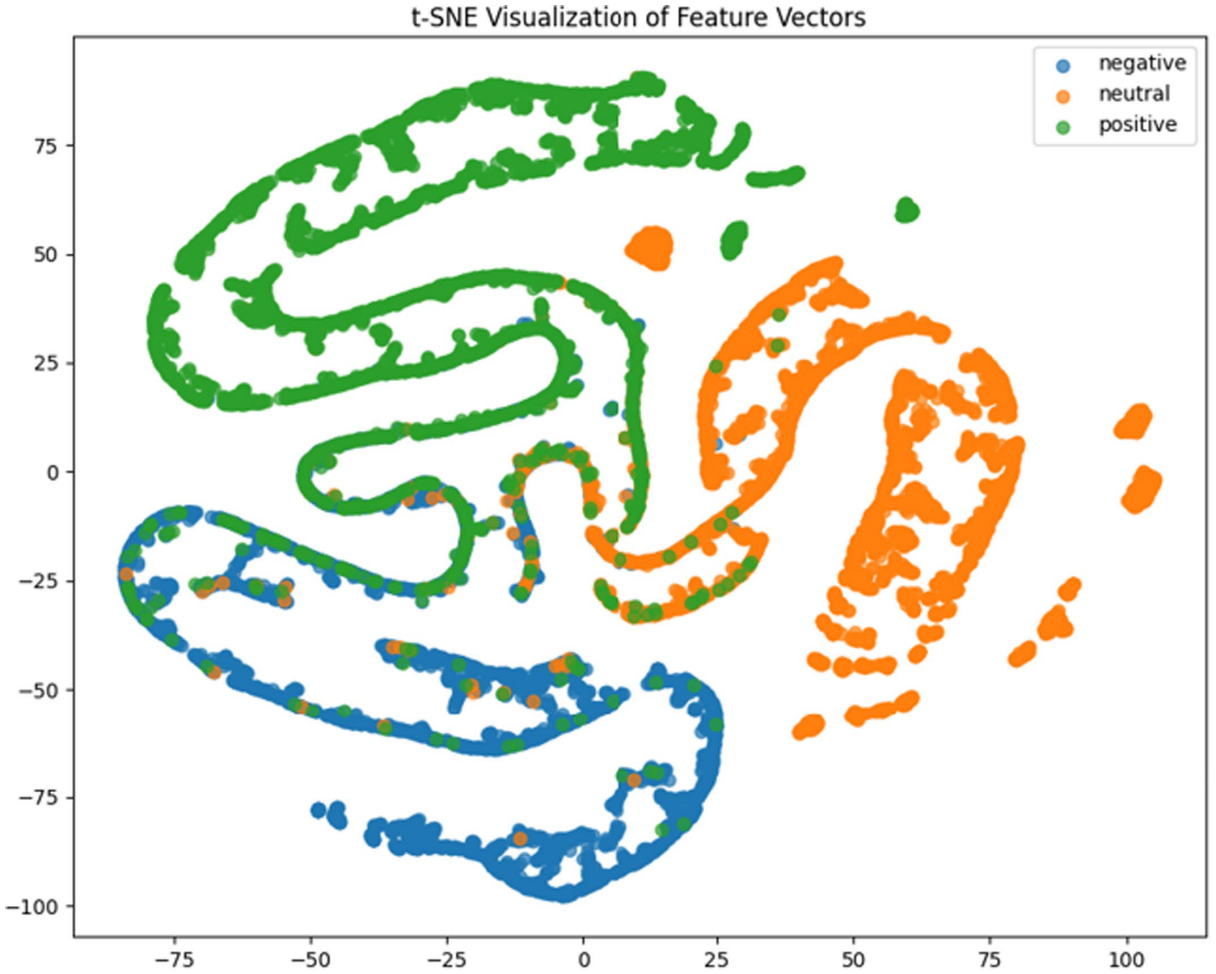
CNN + LSTM t-SNE dimensionality reduction diagram.

**Figure 7:**
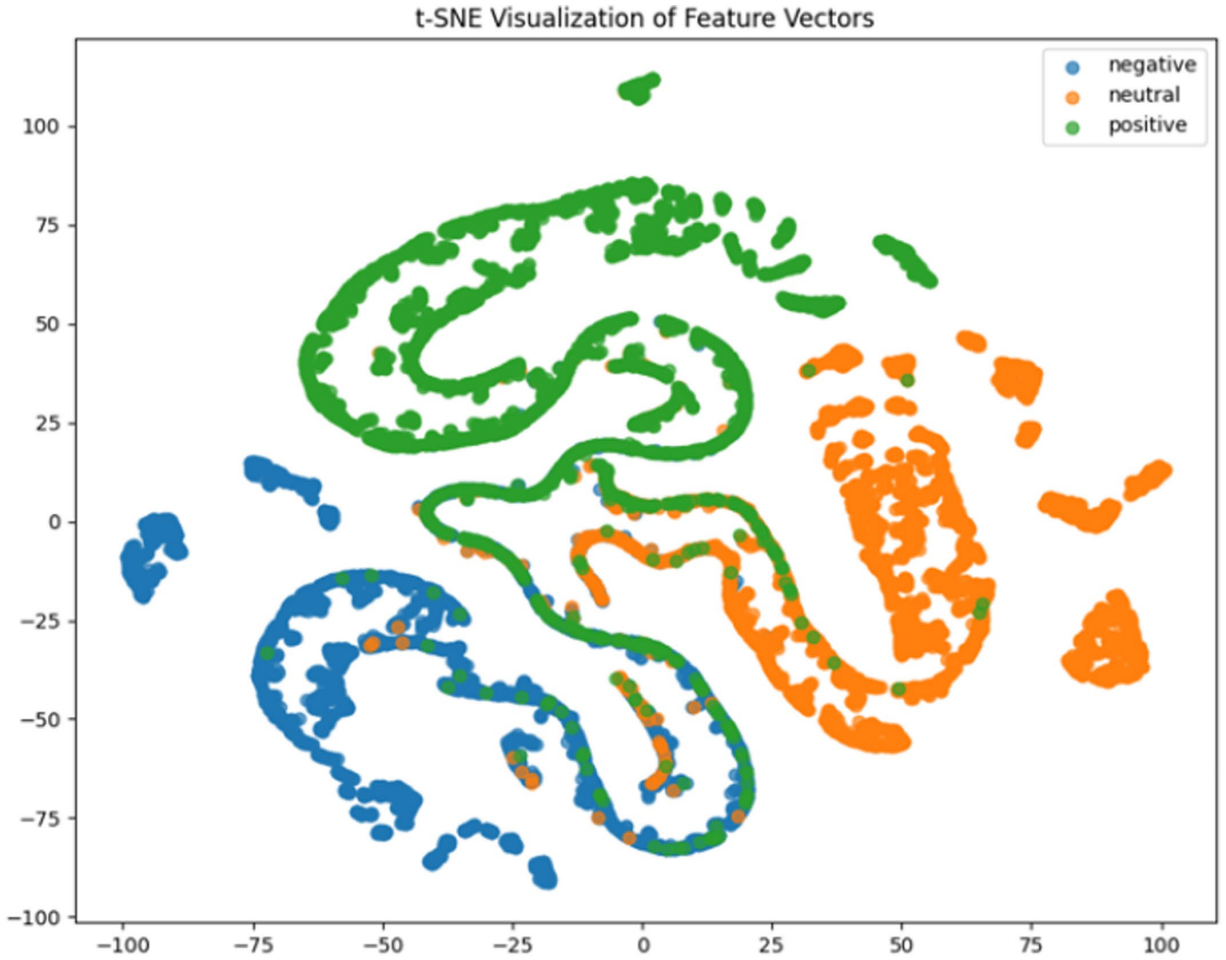
CNN + BiLSTM t-SNE dimensionality reduction diagram.

**Figure 8:**
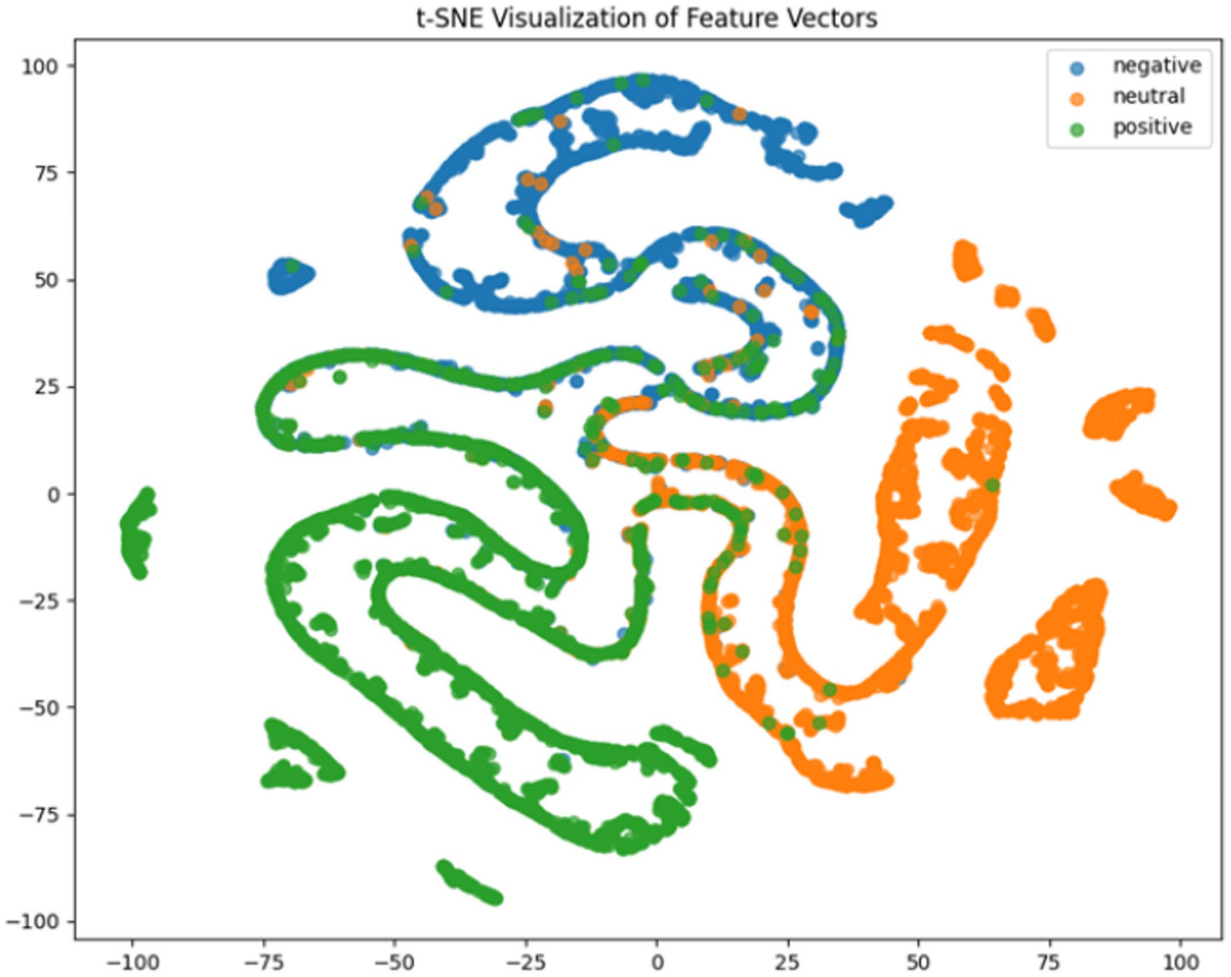
CNN + BiGRU t-SNE dimensionality reduction diagram.

**Figure 9:**
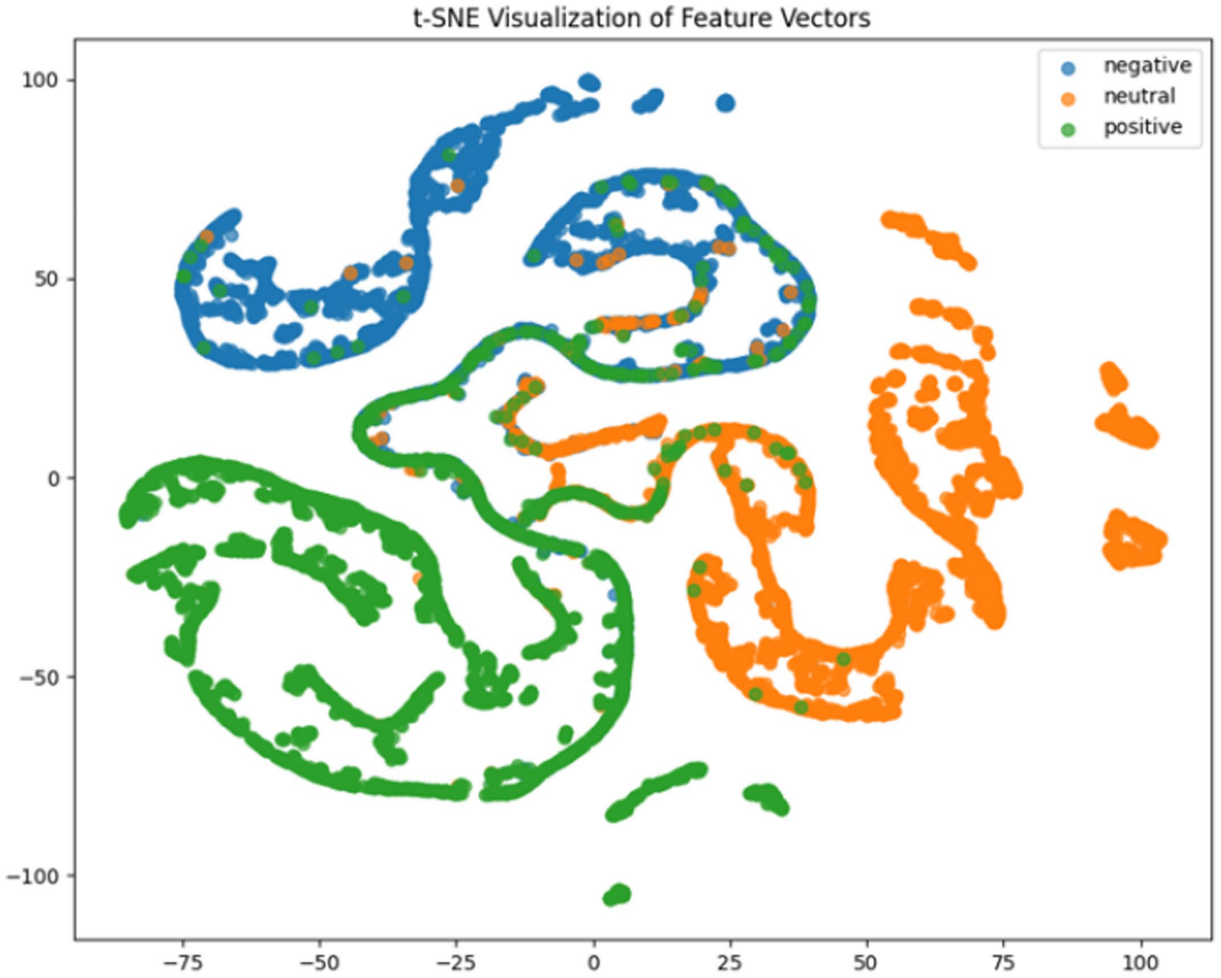
CNN + LSTM+DC-IGN t-SNE dimensionality reduction diagram.

**Figure 10:**
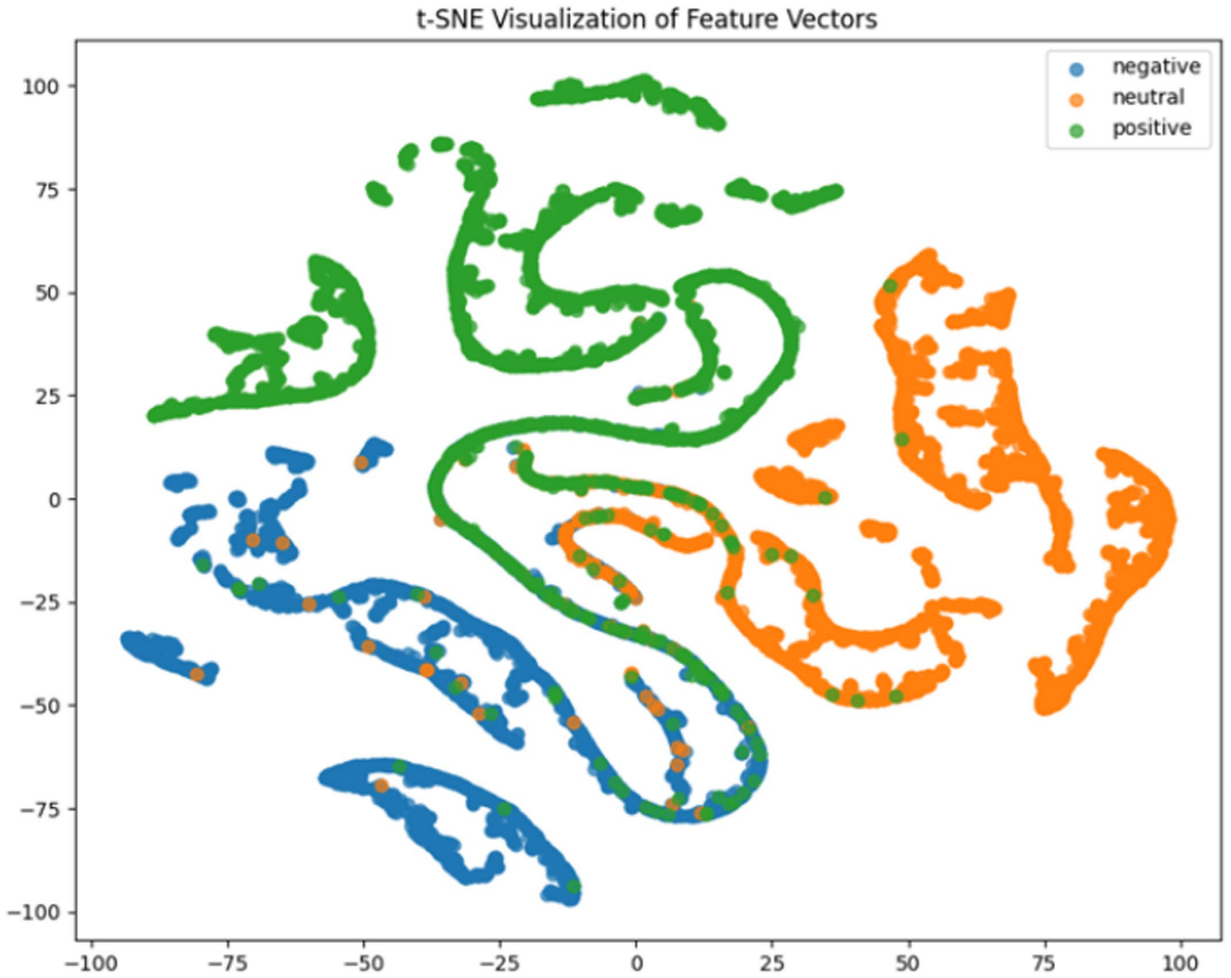
CNN + BiLSTM+DC-IGN t-SNE dimensionality reduction diagram.

**Figure 11:**
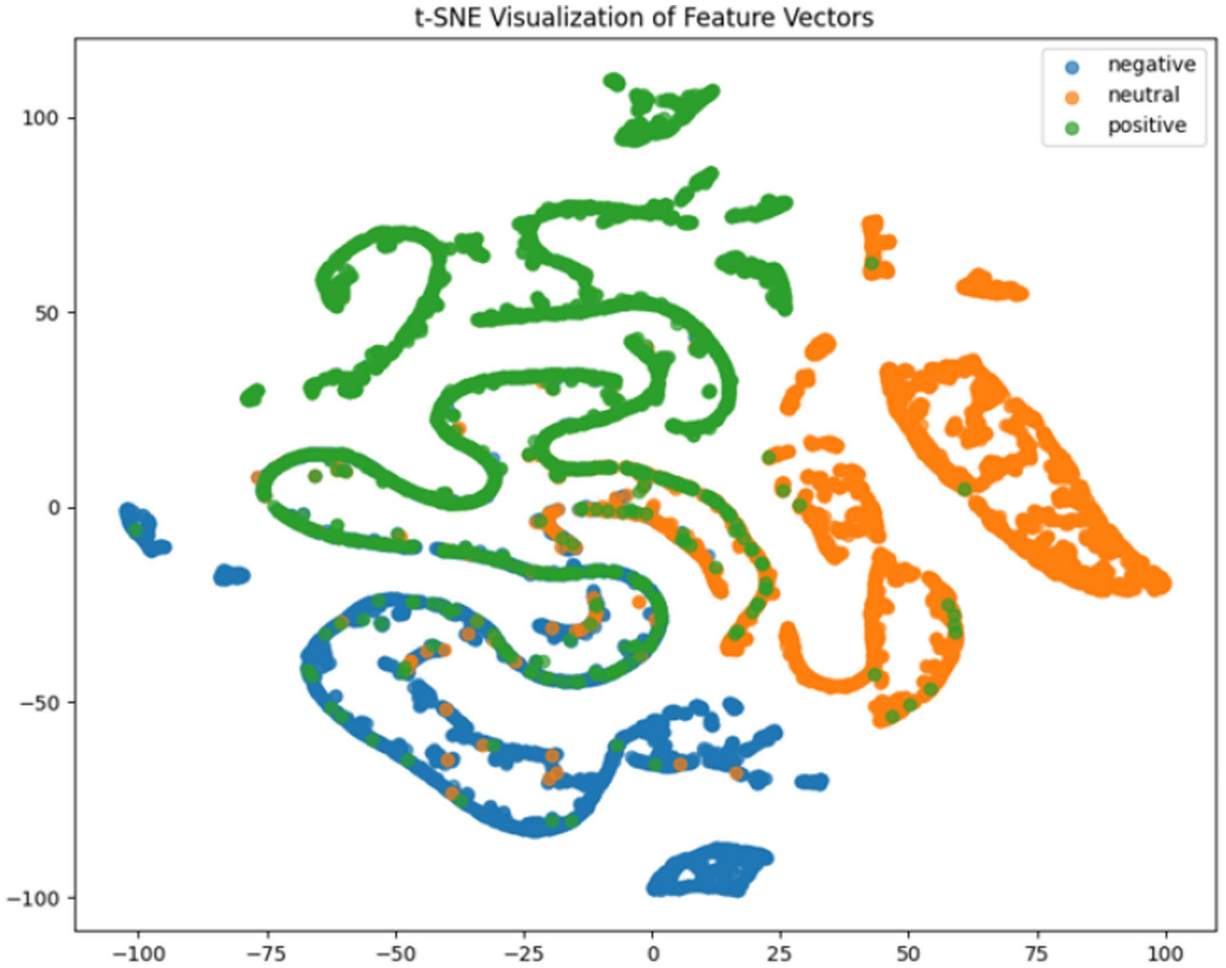
CNN + BiGRU+DC-IGN t-SNE dimensionality reduction diagram.

It can be concluded according to Table 1 and Figures 6–11, the CNN + BiLSTM+DC-IGN model achieves the highest accuracy (94.26%) with the lowest standard deviation (1.34), demonstrating not only superior performance but also the most stable predictions among all evaluated models in the emotion classification task. This exceptional result is attributed to the effective combination and complementary advantages among the model’s components. CNNs excel at extracting local spatial features from EEG data, where each input tensor (shape: 8 × 9 × 4) preserves the spatial and temporal structure of brainwave signals. Through convolutional and pooling operations, CNNs capture critical local patterns for emotion recognition. BiLSTM further enhances temporal modeling by processing bidirectional contextual dependencies in the EEG time series. Unlike unidirectional LSTM, BiLSTM incorporates both historical and future time steps, which is crucial for emotion analysis where states are influenced by temporal dynamics. The high accuracy (92.77%) and low std. (1.53) of the standalone CNN + BiLSTM model already highlight its robustness, but the addition of DC-IGN pushes performance even further. DC-IGN probabilistically models EEG features through its convolutional encoder-decoder architecture, generating richer representations of complex data structures. When integrated with CNN + BiLSTM, DC-IGN contributes additional feature diversity, enabling the fused model to outperform alternatives like CNN + BiGRU+DC-IGN (accuracy: 92.29%, std.: 2.71) or CNN + LSTM+DC-IGN (accuracy: 92.27%, std.: 2.39). The fully connected layer fuses multi-modal features from CNN-BiLSTM and DC-IGN, combining local spatiotemporal patterns with probabilistic representations. This hierarchical fusion improves generalization, as evidenced by the model’s high accuracy and low std. In contrast, less stable models like CNN + BiGRU (std: 3.13) or CNN + LSTM (std: 2.78) exhibit wider performance fluctuations, possibly due to weaker temporal modeling (BiGRU) or unidirectional context (LSTM).

**Table 1:** Comparison of different models on SEED dataset.

model	Accuracy	Std
CNN + LSTM	92.07	2.78
CNN + BiLSTM	92.77	1.53
CNN + BiGRU	90.04	3.13
CNN + LSTM+DC-IGN	92.27	2.39
CNN + BiLSTM+DC-IGN	94.26	1.34
CNN + BiGRU+DC-IGN	92.29	2.71

In summary, the CNN + BiLSTM+DC-IGN architecture leverages the strengths of each component—spatial feature extraction, bidirectional temporal dynamics, and probabilistic feature enrichment—while its minimal std. underscores reliability, making it the optimal choice for emotion classification.

**Figure 12:**
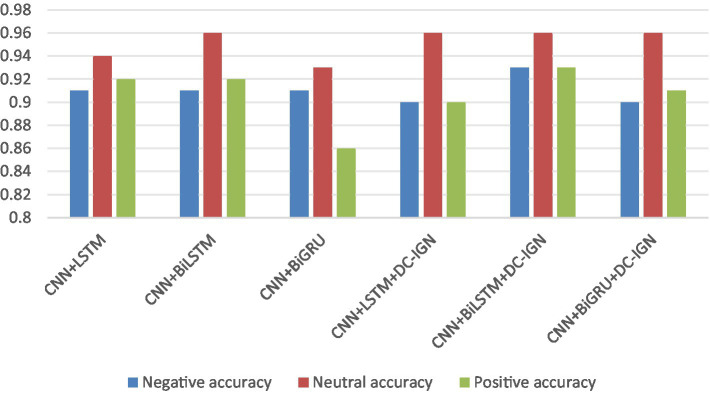
Comparison of three kinds of affective accuracy of different models.

According to the experimental results, the CNN + BiLSTM+DC-IGN model performed best in the emotion classification task, especially in the classification of negative and positive emotions, with an accuracy of 0.93 and 0.93, respectively. This shows that the model can effectively combine the local feature extraction of CNN, the time context modeling of BiLSTM and the probabilistic feature modeling of DC-IGN to comprehensively improve the accuracy of emotion classification when processing multi-channel EEG data. The other models performed better on Neutral emotion, but not as well as the CNN + BiLSTM+DC-IGN model on Positive emotion. This further confirms the importance of multi-component fusion in enhancing sentiment classification performance (Figure 12).

**Figure 13:**
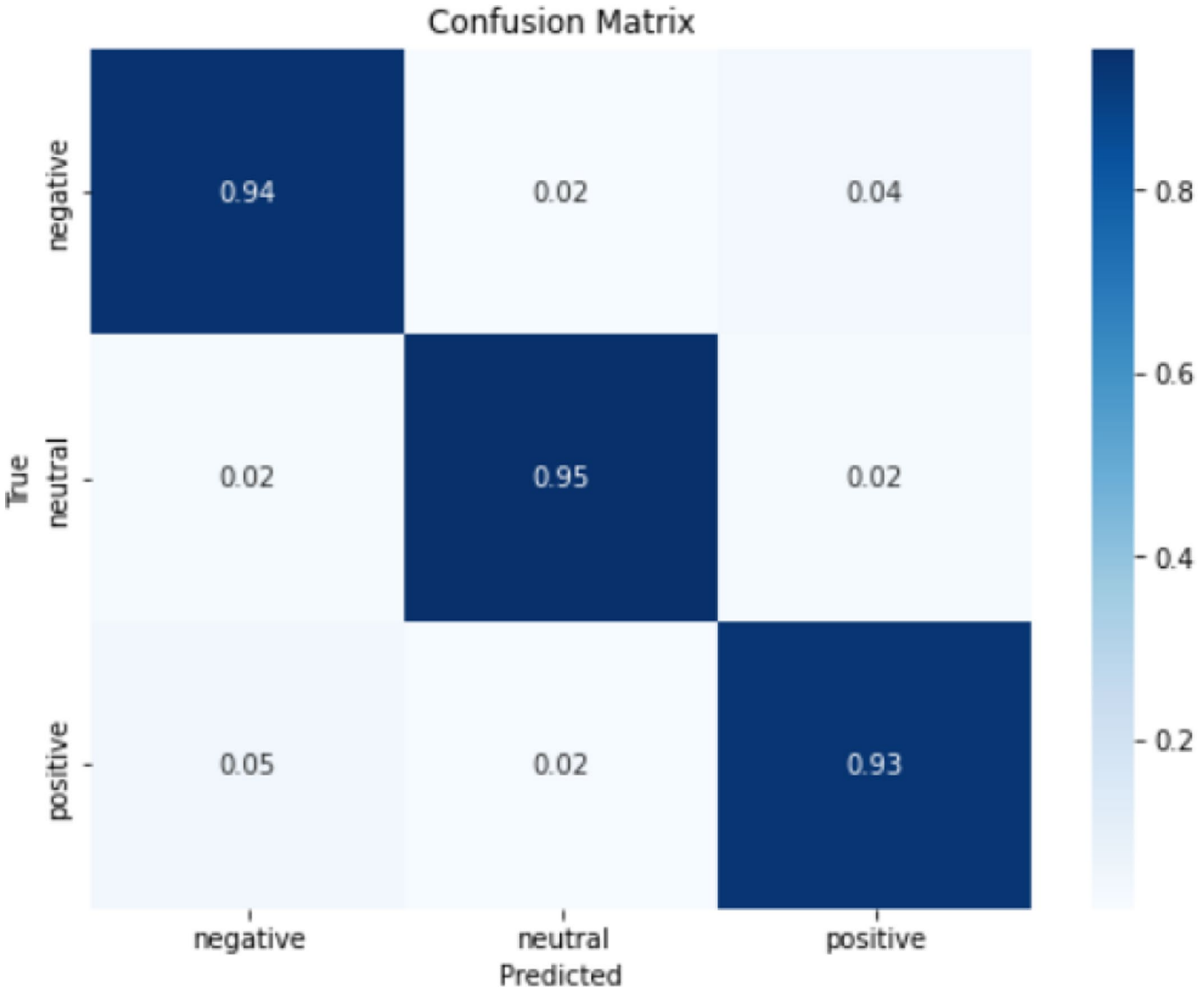
Piecewise exponential attenuation confusion matrix.

It can be concluded according to Table 2 and Figure 13, different learning rate scheduling strategies exhibit varying effects on model performance, both in terms of accuracy and stability. Among the tested strategies, piecewise exponential attenuation achieves the highest accuracy (94.35%) and the lowest standard deviation (1.26), indicating not only superior performance but also the most consistent results across multiple runs. This suggests that the strategy effectively balances convergence speed and training stability. The constant learning rate approach, while simple and intuitive, yields an accuracy of 94.26% with a std. of 1.34. The relatively high std. implies greater variability in model performance, likely due to the fixed learning rate’s inability to adapt to different training phases. This can lead to instability, particularly when gradients are large, increasing the risk of suboptimal convergence. Cosine annealing, despite its theoretical advantage of escaping local optima through periodic learning rate fluctuations, shows the highest std. (1.75) alongside an accuracy of 94.24%. The large variability suggests that the strategy’s aggressive learning rate oscillations may introduce instability, making it less reliable in some training scenarios. Stepped down descent (94.19%, std. = 1.43) and linear descent (94.14%, std. = 1.42) both exhibit moderate accuracy and comparable std. values, indicating consistent but suboptimal performance. The stepwise reduction in learning rate (stepped descent) lacks flexibility, potentially causing abrupt adjustments that hinder fine-tuning. Meanwhile, linear descent’s gradual reduction may slow convergence too early, limiting final accuracy.

**Table 2:** Comparison of learning rate strategies on SEED dataset.

Comparison of learning rate strategies	Accuracy	Std
Constant learning rate	94.26	1.34
Piecewise exponential attenuation	94.35	1.26
Cosine annealing	94.24	1.75
Stepped down descent	94.19	1.43
Linear descent	94.14	1.42

The piecewise exponential decay strategy outperforms others not only in accuracy (94.35%) but also in stability (std = 1.26), demonstrating its ability to balance rapid early-stage convergence with precise late-stage adjustments. In contrast, constant learning rates and cosine annealing show higher variability, while stepped and linear descent deliver steadier but inferior results. From this, it can be seen that the importance of choosing an adaptive learning rate strategy to optimize the performance and repeatability of model training.

**Figure 14:**
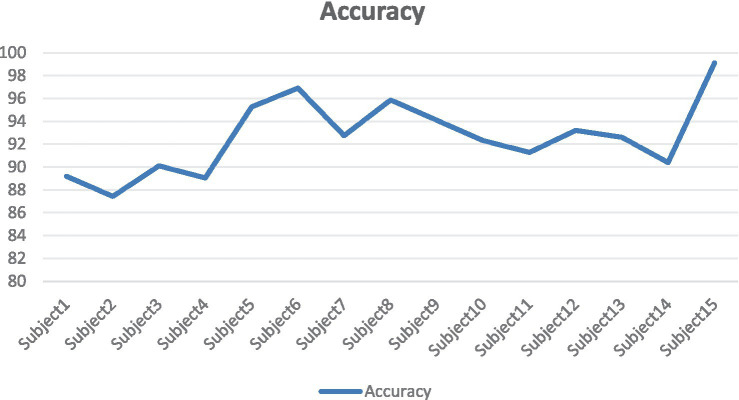
SEED line chart of accuracy for different subjects.

It can be seen from Figure 14 that there are certain fluctuations in the performance of the model on different subjects. The accuracy of Subject15 (99.11%), Subject6 (96.89%) and Subjet8 (95.86%) is significantly higher than that of other subjects. This may indicate that the EEG data features of these subjects are more stable and unique, allowing the model to extract and classify emotional features more efficiently. The accuracy of Subject2 (87.43%) and Subject1 (89.20%) is relatively low. This may be because the EEG data features of these subjects are more complex or noisy, which makes the model difficult to extract and classify. The accuracy of most subjects is concentrated between 90 and 95%, indicating that the model had high robustness and generalization ability in most cases.

Our model achieves 94.35% accuracy (std = 1.26) on SEED dataset, demonstrating both high performance and superior stability compared to other methods. The lower standard deviation of 1.26 indicates more consistent and reliable classification results than BFE-Net (std = 4.65) and ComNet-PSR-VG (std = 4.69), whose higher variations suggest greater performance fluctuations. This stability advantage, combined with our 2.06% accuracy improvement over BFE-Net (92.29%), highlights the robustness of our multi-modal feature extraction approach. Our model’s spatial–temporal-probabilistic feature fusion through CNN-BiLSTM and DC-IGN, coupled with optimized training strategies, not only achieves higher accuracy but also maintains consistently reliable performance across different test cases, addressing the variability issues observed in prior methods. The comprehensive evaluation shows our model outperforms all benchmarks including DGCNN (90.40% ± 8.49) and BiDANN (92.38% ± 7.04), establishing new state-of-the-art performance in EEG emotion recognition (Table 3).

**Table 3:** Comparison with existing models on SEED dataset.

model	Accuracy	Std
GCNN (Defferrard et al., 2016)	87.40	8.64
DGCNN (Song et al., 2020)	90.40	8.49
BiDANN (Li et al., 2021)	92.38	7.04
ComNet-PSR-VG (Yao et al., 2024)	91.39	4.69
BFE-Net (Zhang et al., 2024)	92.29	4.65
Our model	94.35	1.26

The experimental results demonstrate significant improvements in both accuracy and stability after BiLSTM and DC-IGN. Compared to the baseline CNN + LSTM (87.42% ± 8.33 in valence, 88.11% ± 7.98 in arousal), CNN + BiLSTM+DC-IGN achieves superior performance with 88.34% ± 6.45 in valence and 89.55% ± 6.55 in arousal. Notably, the standard deviations consistently decrease across all enhanced models-from 8.33 to 6.45 in valence and from 7.98 to 6.55 in arousal-indicating DC-IGN’s remarkable ability to improve model robustness. The combination of BiLSTM and DC-IGN shows particular effectiveness in arousal classification (89.55% ± 6.55), suggesting its superior capability in capturing complex temporal dynamics and spatial relationships of emotional characteristics. This performance improvement, coupled with reduced variability, confirms the architecture’s enhanced generalization capability for EEG-based emotion recognition tasks (Table 4).

**Table 4:** Comparison of different models on DEAP dataset.

Model	DEAP-valence		DEAP-arousal	
	Accuracy	Std	Accuracy	Std
CNN + LSTM	87.42	8.33	88.11	7.98
CNN + BiLSTM	87.77	8.29	88.69	7.36
CNN + LSTM+DC-IGN	87.46	7.32	89.39	6.91
CNN + BiLSTM+DC-IGN	88.34	6.45	89.55	6.55

When evaluating learning rate strategies on the DEAP dataset, the piecewise exponential attenuation method demonstrated superior performance, achieving 89.84% ± 6.12 in valence and 90.31% ± 6.50 in arousal. This strategy outperforms other approaches not only in accuracy but also in stability, as evidenced by its lower standard deviations compared to most alternatives. The constant learning rate (88.34% ± 6.45, 89.55% ± 6.55) and cosine annealing (86.66% ± 7.14, 90.00% ± 6.86) show competitive performance in arousal but were less stable in valence. The piecewise exponential strategy’s dynamic adjustment capability proves particularly effective for emotion classification, maintaining optimal learning rates throughout different training phases to balance convergence speed and model stability. These results confirm its advantages in handling EEG-based emotion recognition’s complex feature spaces while ensuring robust performance across different emotional dimensions (Table 5).

**Table 5:** Comparison of learning rate strategies on DEAP dataset.

Comparison of learning rate strategies	DEAP-valence		DEAP-arousal	
	Accuracy	Std	Accuracy	Std
Constant learning rate	88.34	6.45	89.55	6.55
Piecewise exponential attenuation	89.84	6.12	90.31	6.50
Cosine annealing	86.66	7.14	90.00	6.86
Stepped down descent	89.53	6.75	88.18	7.92
Linear descent	87.97	7.81	89.22	6.69

**Figure 15:**
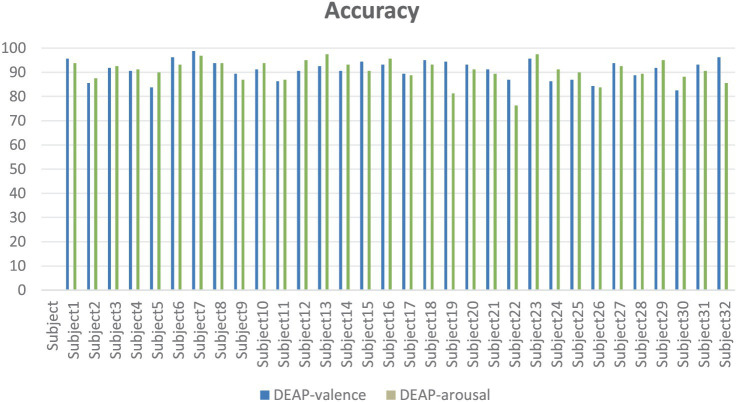
DEAP line chart of accuracy for different subjects.

In experiments on DEAP datasets (Figure 15), our model classifies emotions against EEG data of 32 subjects, and the results show that the model has good generalization ability and stability. On valence and arousal, most of the subjects had an accuracy rate of more than 80 percent, with several of them averaging more than 90 percent. In particular, Subject7 achieved 98.75% and 96.88% accuracy on both tasks, showing the model’s excellent performance when processing specific individual data. Although some subjects, such as Subject22, performed relatively poorly on Arousal task, overall, the model showed a strong ability of emotional recognition. These results further validate the validity of our model in cross-dataset and cross-subject situations, demonstrating its potential for application in the field of affective computing.

Our model shows significant advantages in the emotion recognition task of the DEAP dataset (Table 6), mainly reflected in three aspects: Firstly, an accuracy rate of 90.31% (standard deviation 6.50) is achieved in the arousal dimension, which is 7.8 percentage points higher than the current optimal FLTSDP framework (82.51%), and the stability is better (the standard deviation decreased by 0.78). This breakthrough progress is attributed to our innovatively designed feature fusion mechanism, which can more effectively capture the temporal dynamic features and spatial topological relationships in EEG signals. Secondly, our model is slightly lower than FLTSDP in the dimension of pleasure, and the standard deviation is also slightly lower than FLTSDP, indicating that there is still much room for improvement in our model. This difference may stem from: (1) FLTSDP, which adopts the teacher-student framework, is particularly good at handling static features, while pleasure recognition relies more on stable spatial features; (2) Our dynamic feature extraction mechanism is more sensitive to temporal changes, which gives it an advantage in the wake-up task. Finally, compared with benchmark models such as CLSTM and RACNN, our model has significantly improved in both accuracy and stability (pleasure has increased by 9–15 percentage points, and arousal has increased by 15–22 percentage points), indicating that our improved network architecture can better balance the relationship between spatial feature extraction and time series modeling. Especially, the model maintains a low standard deviation of about 6% in cross-subject scenarios, proving that it has excellent generalization performance, which is of great significance for practical applications. Overall, our model achieves better stability and generalization ability while maintaining a high accuracy rate through innovative network design and feature fusion strategies.

**Table 6:** Comparison with existing models on DEAP dataset.

Model	DEAP-valence		DEAP-arousal	
	Accuracy	Std	Accuracy	Std
CLSTM (Li et al., 2016)	79.21	14.17	68.85	9.61
RACNN (Cui et al., 2020)	80.55	12.50	74.64	8.72
ATDD-LSTM (Du et al., 2022)	74.73	13.07	67.44	8.03
FLTSDP (Gu et al., 2023)	92.40	5.20	82.51	7.28
Our model	89.84	6.12	90.31	6.50

## 5 Conclusion

In this study, an EEG emotion recognition model combining CNN-BiLSTM and DC-IGN is proposed, and the output is fused through the fully connected layer. The experimental results show that the accuracy of the model is 94.35% on SEED dataset, 89.84 and 90.31% on the emotion dimension of DEAP dataset, respectively, which is significantly better than the traditional model and the existing advanced model. The superiority of the model is further verified by subject independent experiment and learning rate scheduling strategy comparison experiment. This study not only improves the performance of EEG emotion recognition, but also provides new research ideas and methods for related fields.

## Data Availability

The original contributions presented in the study are included in the article/supplementary material, further inquiries can be directed to the corresponding author.
